# Protein- and Cell-Resistance of Zwitterionic Peptide-Based Self-Assembled Monolayers: Anti-Biofouling Tests and Surface Force Analysis

**DOI:** 10.3389/fchem.2021.748017

**Published:** 2021-10-06

**Authors:** Ryongsok Chang, Evan Angelo Quimada Mondarte, Debabrata Palai, Taito Sekine, Aki Kashiwazaki, Daiki Murakami, Masaru Tanaka, Tomohiro Hayashi

**Affiliations:** ^1^ Department of Material Science and Engineering, School of Materials and Chemical Technology, Tokyo Institute of Technology, Yokohama, Japan; ^2^ Institute for Materials Chemistry and Engineering, Kyushu University, Fukuoka, Japan; ^3^ Department of Applied Chemistry, Graduate School of Engineering, Kyushu University, Fukuoka, Japan; ^4^ JST-PRESTO, Saitama, Japan; ^5^ The Institute for Solid State Physics, the University of Tokyo, Chiba, Japan

**Keywords:** anti-biofouling behavior, surface force analysis, self-assembled monolayers, blood compatibility, zwitterionic peptide, biocompatibility, atomic force microcopy, quartz crystal microbalance

## Abstract

Peptide-based self-assembled monolayers (peptide-SAMs) with specific zwitterionic amino acid sequences express an anti-biofouling property. In this work, we performed protein adsorption and cell adhesion tests using peptide-SAMs with repeating units of various zwitterionic pairs of amino acids (EK, DK, ER, and DR). The SAMs with the repeating units of EK and DK (EK and DK SAMs) manifested excellent bioinertness, whereas the SAMs with the repeating units of ER and DR (ER and DR SAMs) adhered proteins and cells. We also performed surface force measurements using atomic force microscopy to elucidate the mechanism underlying the difference in the anti-biofouling property. Our measurements revealed that water-induced repulsion with a range of about 8 nm acts between EK SAMs (immobilized on both probe and substrate) and DK SAMs, whereas such repulsion was not observed for ER and DR SAMs. The strength of the repulsion exhibited a clear correlation with the protein- and cell-resistance of the SAMs, indicating that the interfacial water in the vicinity of EK and DK SAMs is considered as a physical barrier to deter protein and cells from their adsorption or adhesion. The range of the repulsion observed for EK and DK SAMs is longer than 8 nm, indicating that the hydrogen bonding state of the interfacial water with a thickness of 4 nm is modified by EK and DK SAMs, resulting in the expression of the anti-biofouling property.

## Introduction

Biomolecules including proteins, lipids, DNA, and polysaccharides smartly interact or react with each other, and then our bodies can maintain the homeostasis of our lives. The biomolecules are responsible for many biomolecular processes such as signal processing, enzymatic reaction, self-assembly, replication. These processes occur in crowding conditions, in which the biomolecules coexist at high concentrations (approximately 200–320 mg/ml in cells) (Cayley et al., 1991). Therefore, biomolecules are endowed with the capability of forming specific bonds with their target molecules and of rejecting other non-target molecules.

To elucidate the mechanism underlying the rejection of non-specific interaction in the molecular recognition of proteins, White *et al.* investigated the composition of amino acid residues at the protein surfaces, which are loca for the biomolecular processes. Their most significant finding was that glutamic acid (E) and lysine (K) are the most and second abundant amino acids (they investigated 1,162 proteins constituting a human body). Moreover, the authors also reported that the zwitterionic pair of E and K is the most frequently found pair on the protein surfaces, followed by DK (D stands for aspartic acid) (White et al., 2012).

Based on these findings, Jiang *et al.* constructed peptide-based self-assembled monolayers (peptide-SAMs) with various repeating zwitterionic pairs of amino acids (Chen et al., 2009; Nowinski et al., 2012; White et al., 2012; White et al., 2013). They found out that self-assembled monolayers (SAMs) containing the repeating units of EK and DK (denoted as EK and DK SAMs hereafter) exhibited excellent anti-biofouling properties, whereas SAMs containing the repeating units of ER and DR (denoted as ER and DR SAMs hereafter) did not. They explained that the difference in the anti-biofouling behavior originates in the different hydration states of the peptide molecules. Unfortunately, the mechanism underlying the sharp contrast in the anti-biofouling properties and the difference in the interfacial interaction have not been clarified experimentally.

This work aims to clarify the interfacial interaction responsible for the protein- and cell-(peptide-SAM) interactions. For this, we first characterize the fundamental physicochemical properties (molecular packing density, thickness, and water contact angle) of the peptide-SAMs and evaluate their anti-biofouling properties by protein-adsorption and cell-adhesion tests. In addition, we performed surface force measurements to elucidate the interfacial interaction responsible for the anti-biofouling property of the peptide-SAMs.

## Materials and Methods

### Fabrication of Peptide-Self-Assembled Monolayers

We used a p-type silicon wafer [Si(100), Thickness and diameter of the wafers are 525 ± 25 μm and 100.0 ± 0.5 mm, respectively, Furuuchi Chemical Co., Japan] cut into pieces with a size of 10 × 10 mm^2^ as the substrates. The silicon substrates were washed by ultrasonic cleaning in acetone, ethanol, and pure water, followed by a nitrogen gas blow.

Colloidal probes were prepared by attaching a washed silica bead (diameter: 18 μm, Duke Sci. Corp., CA, United States) to the end of a tipless cantilever (NP-OW, Bruker, MA, United States) using epoxy glue (Araldite, Huntsman Corp., UT, United States). The nominal spring constant of the cantilevers was 0.06 N m^−1^. After attaching the silica bead, the probes were kept for 12 h at room temperature to fix the bead at the cantilever. Then, the colloidal probes and the silicon substrates were cleaned with a UV-O_3_ cleaner (UV-300, SUMCO, Japan) for 15 min. First, the colloidal probes and substrates were coated with a wetting layer (adhesion promoter) of germanium (99.999%, Nilaco Corp., Japan) with a thickness of 5 nm by thermal evaporation under vacuum (base pressure 1.0 × 10^–5^ Pa) at 350 K. Then, the germanium-coated probes and substrates were coated with gold (99.999%, Furuuchi Chemical Co., Japan) with a thickness of 100 nm at 350 K (Logeeswaran VJ et al., 2009). The deposition rates for germanium and gold were 0.01 and 0.1 nm s^−1^, respectively.

The gold-coated probes and substrates were immersed in a phosphate-buffered saline (PBS) solution (pH 7.4 and ionic strength 167 mM) containing 0.14 mM peptide for 24 h to form covalent bonds between the thiol groups and gold. After the immersion, the probes and substrates were rinsed with pure water to remove excess adsorbed molecules. The peptide-SAMs were prepared for static water contact angle measurement, platelet adhesion test, and surface force measurement. The amino acid sequences of the peptide were summarized in Table 1.

**TABLE 1:** List of peptides used in this work.

Abbreviation	Amino acid sequence
EK	EKEKEKE-PPPPC-Am^a^
DK	DKDKDKD-PPPPC-Am
ER	ERERERE-PPPPC-Am
DR	DRDRDRD-PPPPC-Am

a Am stands for amide bond group (CONH_2_).

### X-Ray Photoelectron Spectroscopy

X-ray photoelectron (XP) spectra in C1s and S2p were measured with a commercial XPS system (Theta Probe, Thermo Electron, United Kingdom). Al Kα radiation (photon energy of 1,486.6 eV and spot size of 400 µm) was used for the X-ray. All XP spectra were acquired at a take-off angle of 48° from the surface normal. The numbers of scans of the C1s and S2p spectra were 40 and 1,000, respectively.

### Static Water Contact Angle Measurement

Static water contact angles (WCA) were measured by the sessile drop method at room temperature (Model G-1-1,000, ERMA, Tokyo, Japan). 2 µl of the water droplet was placed on a peptide SAM, and the WCAs were recorded after 30 s. The measurement was carried out at five different points per substrate using three substrates for one peptide SAM and averaged (n = 15).

### Fibrinogen Adsorption Test

We employed quartz crystal microbalance with an energy dissipation system (QCM-D) (D300, Q-Sense, Sweden) to measure the amounts and viscoelasticity of fibrinogen adsorbed onto the peptide-SAMs. Firstly, gold-coated QCM sensors were cleaned with a UV-O_3_ cleaner for 10 min, then immersed into a 1:1:5 solution of hydrogen peroxide (30%), ammonia (25%), and pure water heated to a temperature of 75 C for 5 min. Immediately rinse with pure water and blow of nitrogen gas. Then the gold-coated QCM sensors were cleaned with UV-O_3_ cleaner for 10 min. The peptide-SAMs were prepared in the same manner for the gold-coated substrates. We used human fibrinogen (Biogenesis Ltd., United Kingdom) dissolved in PBS at a concentration of 1 mg mL^−1^. In the QCM measurements, a measurement chamber was first filled with PBS. Then, the fibrinogen solution was injected into the chamber. After the sensor’s resonant frequency became constant, the PBS solution was injected into the chamber again for rinsing. The amount of the adsorbed fibrinogen was defined as the difference in weights before the injection and after rinsing. The conversion from resonant frequency to weight was based on the Sauerbrey equation shown as Eq. 1,

$$\Delta m=-\frac{C\cdot \Delta f}{n}$$ (1)

where *C* = 17.7 ng cm^−2^ Hz^−1^, Δ*f* is the change in the resonant frequency due to protein adsorption, and the *n* is the overtone number [(*n* = 3) in this work]. All data were collected at the *n* = 3 overtone. The energy dissipation factor *D* was measured by switching off the driving power and monitoring the amplitude decay profile. The amplitude decays as an exponentially damped sinusoidal function with a characteristic decay time (*τ*_0_). The decay time is related to the energy dissipation factor (*D*) shown as Eq. 2,

$$D=\frac{1}{\pi f\tau_{0}}$$ (2)

where *f* is the resonant frequency of the sensor. The viscoelasticity of the protein layer was compared with the value of Δ*d* (change in energy dissipation)/Δ*f* (change in resonant frequency). In this approach, a rigid layer yields a small value of Δ*d*/Δ*f.*

### Platelet Adhesion Test

Blood was drawn from healthy volunteers and mixed with a 1/9 volume of acid citrate dextrose (ACD). Platelet-rich plasma (PRP) and platelet-poor plasma (PPP) were obtained by centrifugation of the blood at 1,500 rpm for 5 min and at 4,000 rpm for 10 min, respectively. The platelet suspension plasma containing 2 × 10^5^ cells µl^−1^ of platelet was prepared by mixing the PRP with PPP. The platelet concentration was determined with a cell-counting hemato-cytometer (Neubauer chamber). Then, 200 µl of the plasma was placed on the peptide-SAMs and incubated for 60 min at 37 C. After the peptide-SAMs were washed with PBS solution twice, the peptide-SAMs were immersed in 1% glutaraldehyde in PBS solution overnight at 4 C to fix the adhered platelets. The fixed samples were immersed in PBS for 10 min, in a 1:1 mixture of PBS and pure water for 8 min, and in pure water for 8 min twice and dried in air overnight. Then the samples were sputter-coated using gold (JFC-1200, JEOL) prior to observation by scanning electron microscopy (VE-9800, KEYENCE, Japan). The number of adhered platelet cells was counted in five positions in the SEM images (100 × 80 μm^2^) using nine substrates for one peptide-SAM and averaged (n = 45).

### Surface Force Measurement

All force curve measurements were performed with a commercial AFM system equipped with a liquid cell (MFP-3D, Oxford Instruments, United Kingdom). Spring constants of the colloidal probes were determined by monitoring the thermal fluctuations of the probes (Hutter and Bechhoefer, 1993). Velocity on approach and retraction of the probe was fixed at 200 nm s^−1^. All force measurements were performed in PBS solution and 1 mM PB (pH 7.4 and ionic strength 2.32 mM) at room temperature. In this study, we simply defined a distance of zero as where linearity in the constant compliance region started in the force-distance curve. We measured 50 times force curves and averaged them.

## Results and Discussion

### Characterization of Peptide-Self-Assembled Monolayers

**FIGURE 1:**
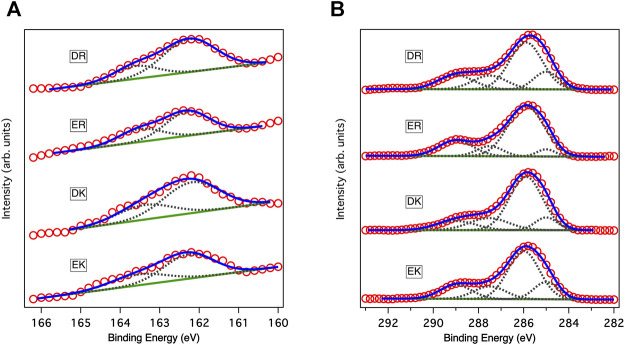
XP spectra of peptide-SAMs in **(A)** C1s and **(B)** S2p regions. Red circles, solid blue lines, solid green lines, and gray dotted lines show data points in the spectra, fitting results, backgrounds, and deconvoluted spectra, respectively. In fitting the spectra in the S2p region, the ratio between the two states and the splitting gap were kept at 2:1 and 1.2 eV, respectively.

Figure 1A shows the XP spectra in an S2p region. The spectra in the S2p region for all peptide-SAMs were deconvoluted into two peaks with an area ratio of 2:1 due to the spin-orbit coupling. The peaks at 162 eV and its satellite peak at 163.2 eV are assigned to the bound state of the sulfur (sulfur is covalently attached to the gold substrate) (Ishida et al., 1999). We did not observe any peaks assigned to the unbound state (usually observed at 163–164 eV), indicating that the peptide molecules are immobilized on gold substrates via a covalent bond and repeating units of the zwitterionic peptides face to the solution phase.

Figure 1B displays the XP spectra of the peptide-SAMs in the C1s region. The spectra are deconvoluted into four peaks. Peaks at about 284, 286, 287, and 289 eV can be assigned to C-(C, H), C-O, C-O next to O=C-O (carbonyl group), and carbonyl group, respectively (Pourcelle et al., 2007). Next, we compared the total peak area divided by the number of carbon atoms in the peptide molecules (Table 2). The results suggest that the peptide molecules are accumulated on the substrate and that the packing densities of DK and DR SAMs are higher than those of EK and ER SAMs. The difference may be attributed to the difference in the steric structure of the amino acid residues in the peptide molecules.

**TABLE 2:** The number of carbon atoms in the peptide molecules and relative packing density with respect to peptides. The packing densities were calculated by C1s intensity and divided by the number of carbon atoms in the molecules.

Peptide-SAMs	Number of carbon atoms in the peptide molecules	C1s intensity/the number of carbon atoms in the molecules
EK SAMs	61	32.0
DK SAMs	57	35.5
ER SAMs	61	31.6
DR SAMs	57	36.3

We also performed force-distance curve measurements using a probe with a sharp tip (tip radius is about 8 nm) (Supplementary Figure S1). The results showed that the thicknesses of the peptide-SAMs were around 2 nm, which corresponds to the Cα-to-Cα end-to-end distance of the EK (White et al., 2012). Taken together, the peptide molecules form a monolayer on gold substrates via covalent gold-sulfur bonds with directing the zwitterionic parts toward the solution phase.

### Fibrinogen Adsorption Test

**FIGURE 2:**
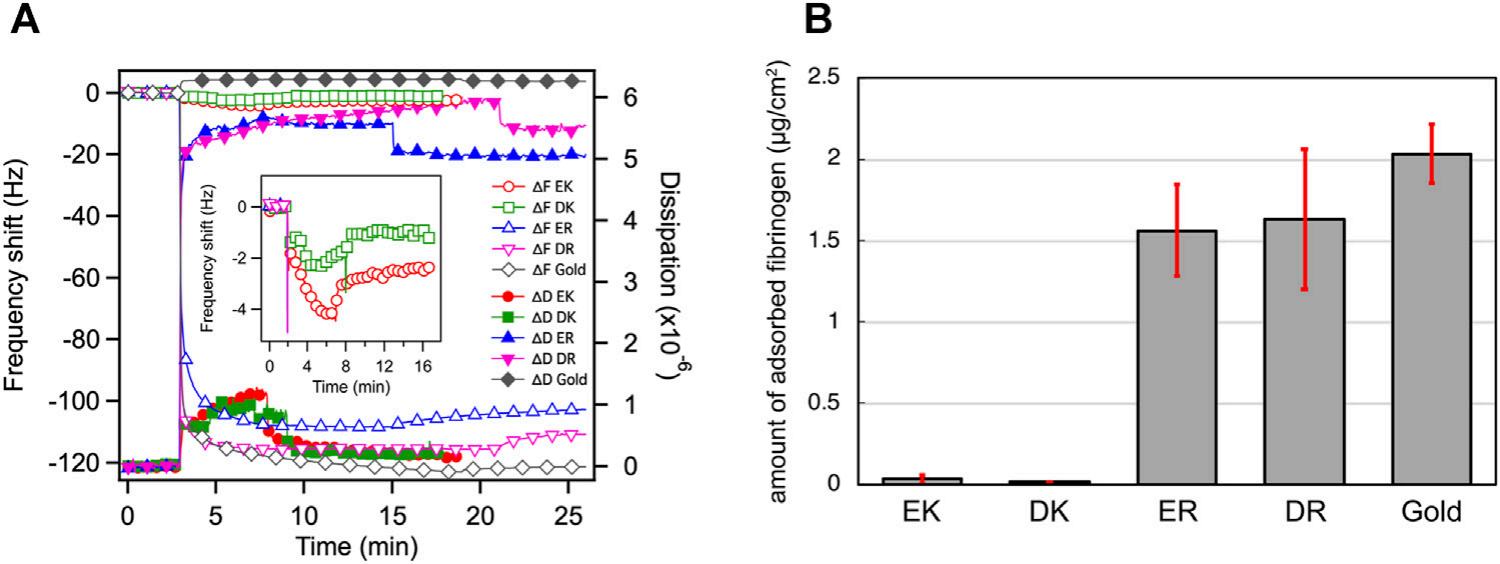
**(A)** QCM chart (frequency and dissipation) observed for adsorption of fibrinogen onto the peptide-SAMs. Rinsing with PBS were performed at 8, 9, 15, 21, 17 min for EK, DK, ER, DR, and bare gold, respectively. **(B)** The amount of adsorbed fibrinogen on peptide-SAMs in the PBS solution measured by QCM-D. Error bars denote standard deviation (*n* = 3).

Figure 2A shows the change in the resonant frequency and energy dissipation measured in the QCM measurements. ER and DR SAMs adsorbed fibrinogen with a significant increase in the dissipation rapidly after the injection of the protein solution, whereas we did not observe the adsorption for EK and DK SAMs without a drastic change in the energy dissipation. Figure 2B compares the amount of the adsorbed fibrinogen after rinsing, displaying the clear contrast in the degree of protein resistance, in good agreement with the report by Chen et al. (2009). In particular, the protein-resistance of EK and DK SAMs are comparable to that of the SAMs comprised of oligo (ethylene glycol)- or sulfobetaine-terminated alkanethiols, which are often widely used for the suppression of non-specific adsorption in biosensing (Hayashi et al., 2012; Tanaka et al., 2013; Sekine et al., 2015; Chang et al., 2018; Kwaria et al., 2020; Hayashi, 2021; Tanaka et al., 2021).

The Δ*d*/Δ*f* values also provide insight into the mechanical properties of the protein layer formed on the peptide-SAMs (Table 3). The Δ*d*/Δ*f* values for protein-resistant EK and DK are higher than those for protein-adsorbing ER and DR SAMs (Table 3). These results indicate that the interaction of fibrinogen with ER and DR SAMs is stronger than that with EK and DK SAMs and/or the formed protein layer is highly viscoelastic because of the denaturation of the protein molecules after adsorption.

**TABLE 3:** Δ*d*/Δ*f* of fibrinogen on the peptide-SAMs in the PBS solution measured by QCM-D after the rinsing. Numbers in parenthesis are standard deviations (n = 3).

Peptide-SAMs	Δ*d*/Δ*f* (10^–6^)
EK SAMs	0.25 (0.078)
DK SAMs	0.13 (0.046)
ER SAMs	0.04 (0.006)
DR SAMs	0.04 (0.005)

### Platelet Adhesion Test

**FIGURE 3:**
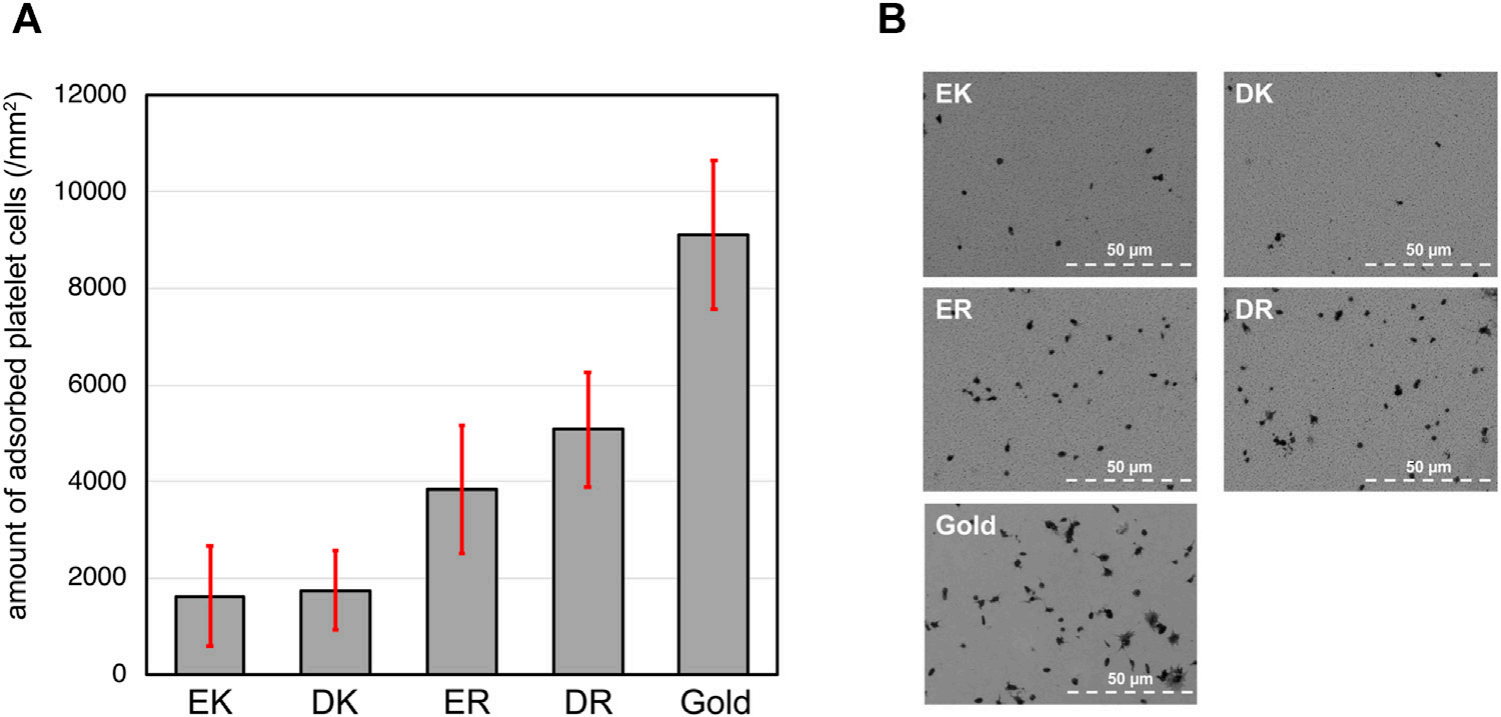
**(A)** The density of adhered platelet cells on peptide-SAMs and gold surface. Error bars denote standard deviation (n = 45). **(B)** Representative SEM images of the adhered platelet cells on the peptide-SAMs and the gold surface. Note that the particles smaller than the platelets are gold clusters formed after the deposition of gold to enhance the conductivity for SEM imaging.

The density of adhered platelet cells on the peptide-SAMs and the representative SEM images of the substrates are summarized in Figures 3A,B. Compared with the bare gold substrates, the adhesion and activation of platelets were suppressed on the peptide-SAMs. However, there was a distinct difference in their anti-platelet adhesion properties. Apparently, we can conclude that the platelet compatibility of EK and DK SAMs is higher compared with ER and DR SAMs.

In our platelet adhesion test, plasma proteins first accumulate on the peptide-SAMs and form a layer. Then, the protein layer interfaces the peptide-SAMs and platelet cells. The proteins’ composition, orientation, and conformation significantly affect the adhesion and activation of platelet cells (Hylton et al., 2005). There have been many works reporting a clear correlation between protein adsorption and platelet adhesion. In particular, adsorption tests of fibrinogen correlate well with platelet cells’ adhesion (Tanaka et al., 2000). Our results here are in good agreement with previous findings. The analysis of the protein composition by mass spectroscopy is underway (Hirohara et al., 2019).

### Static Water Contact Angle Measurements

Water contact angles (WCA) have often been an indicator of protein- and cell resistance for monolayer and polymer systems, although there are apparent exceptions (Alexander and Williams, 2017; Kwaria et al., 2020). We also investigated the surface wettability of peptide-SAMs by WCA on the peptide-SAMs (Table 4). The WCA showed that all peptide-SAMs are hydrophilic in terms of macroscopic wettability, indicating that strong interaction of fibrinogen with ER and DR SAMs is due not to hydrophobic interaction but to electrostatic interaction.

**TABLE 4:** Static water contact angles (WCA).

Peptide-SAMs	Static water contact angle^a^
EK SAMs	34 (11)
DK SAMs	27 (9.2)
ER SAMs	29 (8.1)
DR SAMs	34 (11)

a Values in parentheses are standard deviation (n = 15).

The main finding here is that the bioinertness of the zwitterionic peptide-SAMs cannot be explained with the macroscopic wettability of the peptide-SAMs. This has also been found for the SAMs of oligo (ethylene glycol)-terminated and several blood compatible polymers, including relatively hydrophobic poly (2-methoxyethyl acrylate) (PMEA) and hydrophilic poly (2-methacryloyloxyethyl phosphorylcholine) (PMPC) (Hayashi et al., 2007; Tanaka et al., 2013; Hayashi, 2021; Tanaka et al., 2021).

### Surface Force Measurements

**FIGURE 4:**
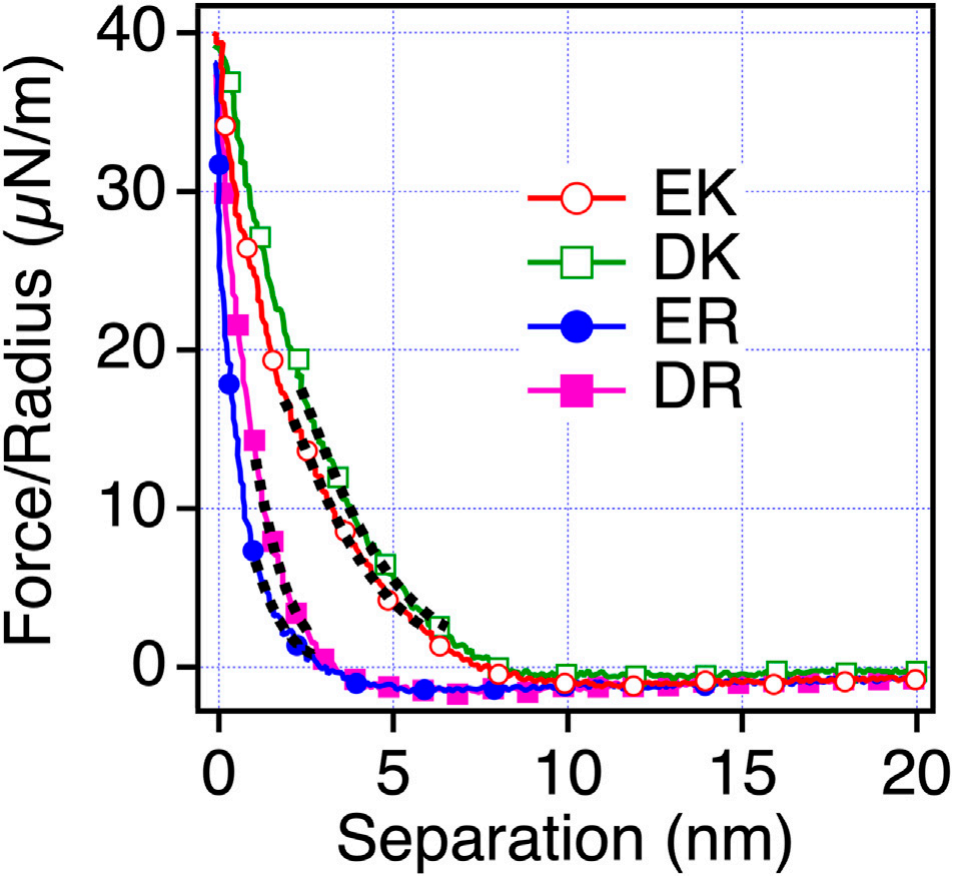
Force-separation curves recorded on an approach for the symmetric system of the peptide-SAMs (the same SAMs formed on both probe and substrate) in PBS solution. The fitting results are shown with black dotted lines.

To understand the mechanism underlying the anti-biofouling property of EK and DK SAMs and the difference in the protein and cell resistance among the peptide-SAMs, we investigated the surface force induced by the peptide-SAMs. To clarify the physical origin of the surface forces, we performed surface force measurements in PBS solution, which is the solution condition used in protein adsorption and cell adhesion tests (Figure 4). The repulsive force was observed for all the peptide-SAMs, although the ranges of the repulsive force are different. As for ER and DR SAMs, the onset of the repulsion is at 4–5 nm of the separation. In contrast, the working distance of the repulsion for EK and DK SAMs is at around 8–9 nm. The repulsion’s decay lengths obtained by fitting the curves with a single exponential function are summarized in Table 5. The interaction in water is described as a sum of DLVO (electrostatic and van der Waals interaction) and non-DLVO (hydration force, steric repulsion, etc.) interactions. Among them, possible origins of the repulsive force are electrostatic double-layer force (DLVO), hydration force, and steric repulsion due to the deformation of the SAM after the physical contact of the peptide-SAMs (non-DLVO). Considering that the thicknesses of all the peptide-SAMs are about 2 nm (Supplementary Figure S1), the steric repulsion is not the origin of the difference in the repulsion observed in the force measurements.

**TABLE 5:** Decay lengths of the repulsion acting between the peptide-SAMs in PBS.

Peptide-SAMs	Decay length in PBS (nm)
EK SAMs	1.76
DK SAMs	1.86
ER SAMs	0.698
DR SAMs	0.746

**FIGURE 5:**
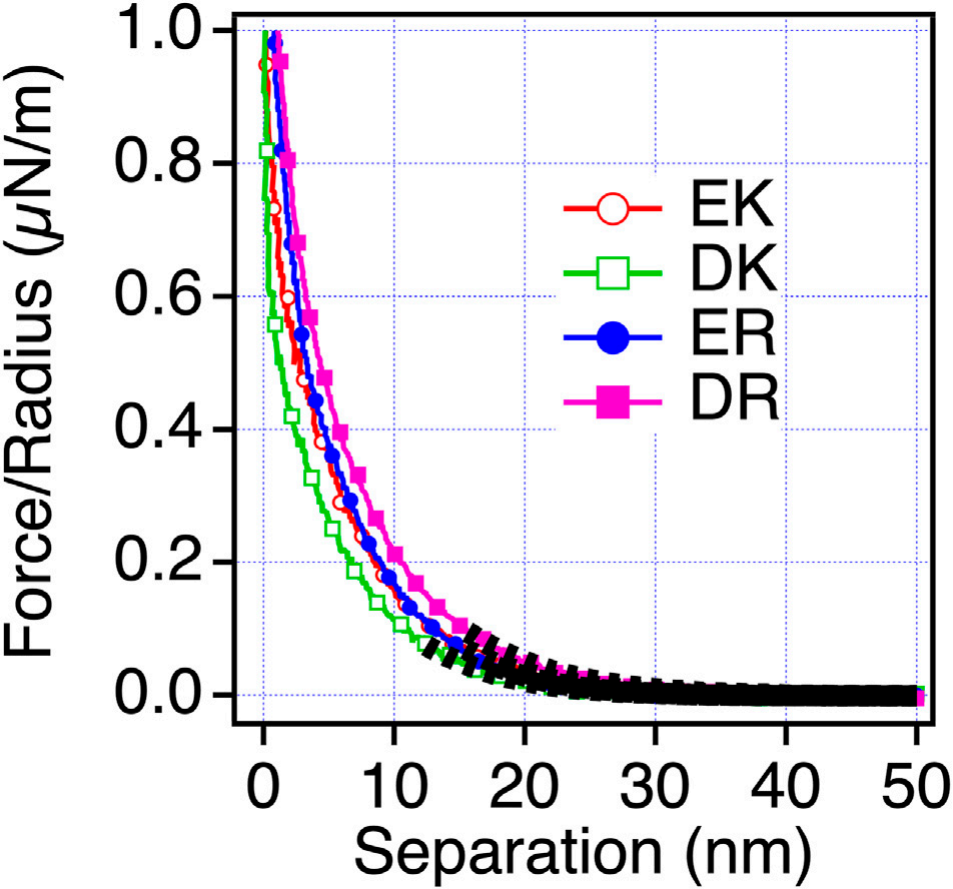
Force-separation curves recorded on an approach for the symmetric system of the peptide-SAMs (the same SAMs prepared for both the probe and substrate) in a 1 mM PB solution. The fitting results are shown in a black dotted line.

Next, we examined the possibility of the electric double layer force for the difference in the repulsion by changing the ion concentration of the solution. We performed the measurements in phosphate buffer (PB) (1 mM) solution, whose Debye length is compared with PBS solution (Figure 5). With this solution condition, we observed the repulsion for all the peptide-SAMs, and the force curves can be fitted with the DLVO theory shown as Eq. 3,

$$F\left(D\right)=-\frac{H_{s}R}{6D^{2}}+\frac{4\pi R\epsilon \epsilon_{0}\psi^{2}}{\lambda_{D}}\left[exp\left(\frac{-D}{\lambda_{D}}\right)-exp\left(\frac{-2D}{\lambda_{D}}\right)\right]$$ (3)

where *H*_*s*_, *D*, *R, ε*, *ε*_0_, *ψ*, and *λ*_*D*_ are the Hamaker constant for the interaction of peptide-SAMs across the water, the separation between the probe and surface, probe radius, relative dielectric constant, dielectric constant of vacuum, the surface potential of the surface and probe (peptide-SAMs), and the Debye length, respectively (Butt et al., 2005). The results of the fitting are shown in Figure 5 and Table 6.

**TABLE 6:** Estimated surface potential and Hamaker constant from force-separation curves between peptide SAMs in 1 mM PB.

Peptide-SAMs	Debye length (nm)	Surface potential (mV)	Hamaker constant (J)
EK SAMs	6.2	8.5	1.2 × 10^–21^
DK SAMs	5.8	7.4	1.0 × 10^–21^
ER SAMs	5.4	9.2	1.2 × 10^–21^
DR SAMs	6.7	9.7	2.4 × 10^–21^

The fitting results show that the calculated Debye lengths range between 5.4 and 6.7 nm. Compared with the decay length observed in PBS, the decay lengths of the repulsion are much longer compared with those in PBS (theoretical Debye length of PBS is 0.745 nm) and are in agreement with the theoretical value (6.32 nm). Therefore, we concluded that electrostatic double-layer force is dominant in PB solution. Unfortunately, we cannot determine the signs of the surface potentials because the same SAMs were formed on both probe and surface (Eq. (3)). By considering the strong basic character of arginine (R) (Fitch et al., 2015), we speculate that ER and DR SAMs are more positively charged than EK and DK SAMs.

The trend in the strength of the repulsion depending on the sequences in PB is different from that in PBS. Therefore, we consider that the repulsion observed for EK and DK SAMs is not electrostatic double-layer force. The decay lengths of the repulsion observed for EK and DK SAMs are far beyond the theoretical Debye length of PBS (0.745 nm) and cannot be explained by the electrostatic double-layer force. Combining the above discussions, the only possible origin for the repulsion is water-induced force.

**FIGURE 6:**
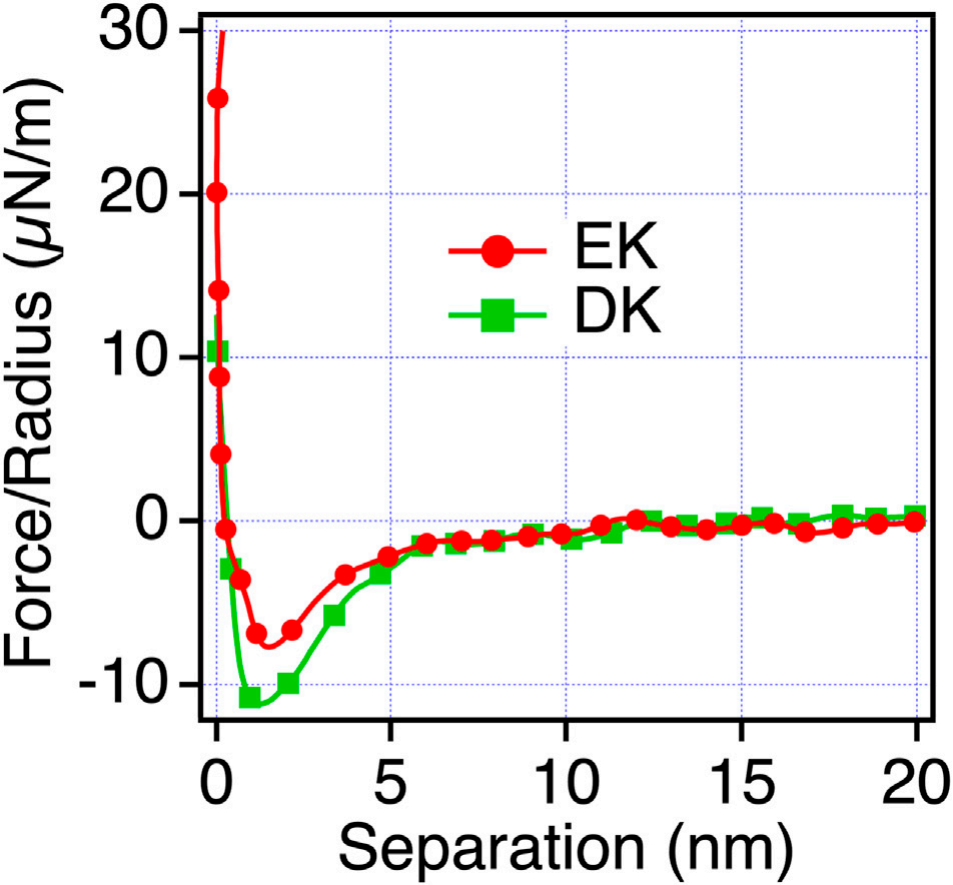
Force-separation curves recorded on an approach for the symmetric system of the EK and DK SAMs (the same SAMs prepared for both the probe and substrate) in ethanol and PBS solution [0.23 mol (ethanol)/mol (water)].

To confirm that the origin of the repulsive force observed for EK and DK in PBS is water-induced force, we measured the interaction force in mixtures of PBS and ethanol. When water is mixed with ethanol, water and ethanol molecules are phase-separated at a low concentration of ethanol. When the concentration of ethanol increases, ethanol molecules are mixed with water molecules with disrupting three-dimensional networks of hydrogen bonding of water. At the ethanol’s molar ratio of 0.23, water and ethanol completely mix together (Matsumoto et al., 1995). As seen in Figure 6, The short-range repulsion in PBS disappeared in ethanol and PBS solution. The results indicated that water molecules near EK and DK possess the hydration barrier with a thickness of 3–4 nm (half of the working distance of the repulsion force) and prevent the approaches of protein molecules and cells, whereas ER and DR possess the hydration barrier with a thickness less than 2 nm and allowed their approaches.

## Conclusion and Perspectives

In this work, we attempted to unveil the mechanism underlying the anti-biofouling properties of zwitterionic peptide-SAMs reported by Chen et al. (2009) and White et al. (2012). First, we investigated the adsorption states of the peptide molecules by XPS and measured the thicknesses of the peptide layers by AFM, confirming that the molecules form monolayers immobilized via covalent Au-S bonds on gold. Second, we verified that EK and DK SAMs exhibited excellent protein- and platelet resistance, whereas ER and DR did not, leading to the conclusion that the difference in the anti-biofouling property cannot be explained by their macroscopic surface wettability.

To evaluate the interfacial interaction responsible for the anti-biofouling property, we performed surface force measurements to assess the interaction between the peptide-SAMs. Water-induced repulsion with a range more extended than 8 nm was observed for EK and DK SAMs, whereas only weak and short-ranged (<4 nm) repulsion was observed. Furthermore, there was a clear correlation of protein- and platelet-resistance with the water-induced repulsion. Therefore, we concluded that the water-induced repulsion induced by the EK and DK SAMs is responsible for their anti-biofouling property.

Our previous surface force measurements revealed a similar water barrier for SAMs of oligo (ethylene glycol)-terminated alkanethiols (OEG-SAMs) and DNA-based SAMs (Hayashi et al., 2012; Sekine et al., 2015; Kanayama et al., 2016; Sekine et al., 2017; Sekine et al., 2018). As for the OEG-SAMs, the strength and acting range of the water-induced repulsion are very sensitive to the number of the EG units in the molecule and molecular packing density. In DNA-based SAMs, the strength and acting range critically depend on the complementarity of terminal base pairs of the DNA molecules. These facts suggest that the local arrangement of functional groups with respect to water modulates, and the resulting hydrogen bonding network of the interfacial water determines anti-biofouling potential.

There have been many works on the hydration of zwitterionic functional groups, including carboxy or sulfobetaine, phosphatidylcholine, zwitterionic peptides. Many of them stated the importance of the strongly trapped water molecules to the charged groups in their anti-biofouling property. Also, some works suggested that the guanidinium group in R is one of the weakly hydrated cations and this may be a weak protein resistance of ER and DR SAMs (Mason et al., 2003; White et al., 2013). However, our surface force analysis revealed that the water barrier extends up to 4 nm, suggesting that the water-peptide (EK and DK) interaction modulates the hydrogen bonding states of the vicinal water molecules, resulting in the formation of the physical barrier to prevent the approach of protein molecules and cells.

In this work, we clarified the correlation among the amino acid sequence of the peptide constituting the SAMs, anti-biofouling property, and water-induced interfacial forces. However, unfortunately, we have not elucidated the molecular processes inducing water-mediated repulsion responsible for the anti-biofouling property. Currently, we are performing surface vibrational spectroscopy measurements and theoretical calculations to understand the hydrogen bonding states of the interfacial water. These results will be published elsewhere.

## Data Availability

The original contributions presented in the study are included in the article/Supplementary Files, further inquiries can be directed to the corresponding author.
