# Supplementary material for: Protein- and Cell-Resistance of Zwitterionic Peptide-Based Self-Assembled Monolayers: Anti-Biofouling Tests and Surface Force Analysis
Source: Front Chem. 2021 Oct 6;9:748017. doi: 10.3389/fchem.2021.748017 (PMC8527039; doi:10.3389/fchem.2021.748017)
Supplement: Supplementary file 1 [file DataSheet1.PDF]

Supplementary Material

Characterization of the SAMs' thicknesses with AFM

All force curve measurements were performed with a commercial AFM system equipped with a liquid cell (MFP-3D, Oxford Instruments, U.K.). A small silicon cantilever with a rectangular shape (BL-AC40TS, Olympus Co., Japan) was used. The nominal curvature radius of the probe is 8 nm. Spring constant of the cantilever was determined by monitoring the thermal fluctuations of the probes.[1] The nominal spring constant is 0.1 N/m. All force measurements were performed in PBS solution at room temperature. Force maps were recorded in a contact mode. Velocity on approach and retraction of the probe was fixed at 3 µm s^{-1} and applying a maximum loading force of 2.5 nN. The resolution was set at 100 × 100 points with a scan area of 1 × 1 μm^2, generating 10000 force curves per force map (10 × 10 nm per 1 point). For conversion of a deflection of the cantilever to the tip-surface separation, we simply defined a distance of zero as where linearity in the constant compliance region started in the force-distance curve. To reveal the thickness value of peptide SAMs, we averaged the 100 force curves.

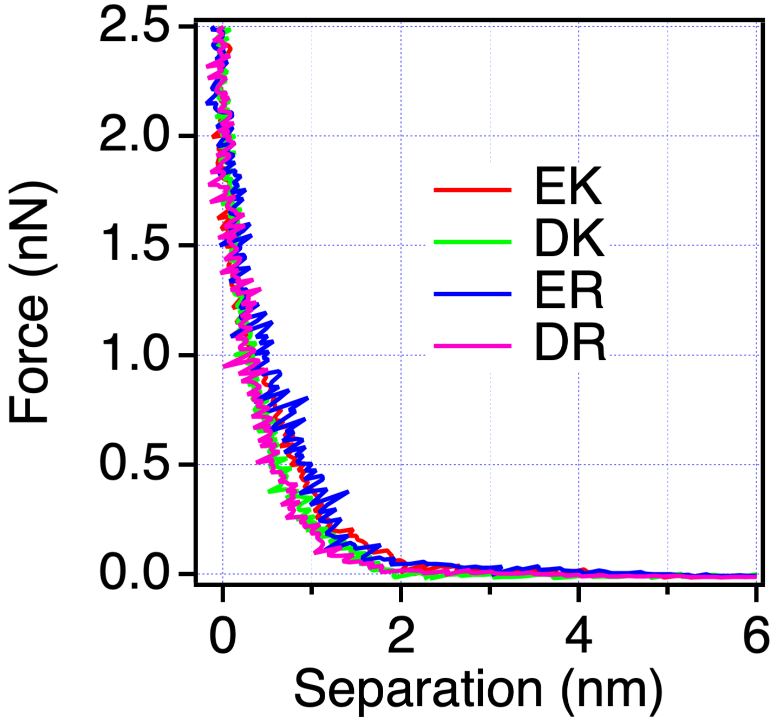

Supplementary Figure 1. Force-separation curves recorded on an approach for peptide SAMs in PBS solution.

References:

[1] Hutter, J.L.; Bechhoefer, J. Calibration of atomic-force microscope tips. *Review of Scientific Instruments* **1993**, *64*, 1868-1873, doi:10.1063/1.1143970.
